# CARDIOVASCULAR HEALTH AND SUCCESSFUL AGING: THE MULTI-ETHNIC STUDY OF ATHEROSCLEROSIS

**DOI:** 10.1093/geroni/igz038.2394

**Published:** 2019-11-08

**Authors:** Tzuo-Yun Lan, Amy Krefman, Michael Bancks, Steven Shea, Kiang Liu, Norrina Allen

**Affiliations:** 1 Institute of Hospital & Health Care Administration, National Yang-Ming University, Taipei, Taiwan; 2 Department of Preventive Medicine, Feinberg School of Medicine, Northwestern University, Chicago, Illinois, United States; 3 Wake Forest University School of Medicine, Winston Salem, North Carolina, United States; 4 Columbia University Mailman School of Public Health, New York, New York, United States

## Abstract

Individual risk factors have been shown to be associated with successful aging. However, the combined effect of behaviors and biomarkers on successful aging remains unclear. By using the Multi-Ethnic Study of Atherosclerosis (MESA) dataset, this study was to examine the association of AHA’s Cardiovascular Health (CVH) with successful aging. A total of 1,597 who were followed from baseline (2000-2002) at age 49-64 through exam 6 (2016-2018) at age 65-80 were included. CVH, including smoking, body mass index (BMI), physical activity, diet, blood pressure, cholesterol, and blood glucose, was measured at baseline. The CVH score, ranging from 0-14, was divided into ideal (11–14), intermediate (9–10), and poor (0–8) groups. Normal or successful aging, defined as avoiding major disease (including cancer, cardiovascular, or severe lung or kidney diseases), no disability, high cognitive function, high physical functioning, and engagement with life, was assessed at exam 6. We compared the cumulative incidence of successful aging among three groups. Modified Poisson regression model was employed to estimate relative risk (RR) adjusting for age, gender, race, education, income, marital status, and alcohol consumption. Among study participants at baseline, 36% were in ideal, 39% in Intermediate, and 25% in poor CVH. By exam 6, only 18% met the criteria for successful aging. Compared with the poor group, the adjusted RRs (95 % CI) of successful aging for the intermediate and ideal groups were 1.78 (1.23-2.56) and 2.56 (1.79-3.67). Our data suggest that CVH in midlife is associated with successful aging in later life.

